# Test–retest, inter- and intra-rater reproducibility of size measurements of focal bone marrow lesions in MRI in patients with multiple myeloma

**DOI:** 10.1259/bjr.20220745

**Published:** 2023-04-12

**Authors:** Markus Wennmann, Martin Grözinger, Vivienn Weru, Thomas Hielscher, Lukas Thomas Rotkopf, Fabian Bauer, Regula Gnirs, Tobias Nonnenmacher, Sandra Sauer, Hartmut Goldschmidt, Niels Weinhold, David Bonekamp, Tim Frederik Weber, Heinz-Peter Schlemmer, Stefan Delorme

**Affiliations:** 1 Division of Radiology, German Cancer Research Center (DKFZ), Heidelberg, Germany; 2 Division of Biostatistics, German Cancer Research Center (DKFZ), Heidelberg, Germany; 3 Medical Faculty, University of Heidelberg, Heidelberg, Germany; 4 Diagnostic and Interventional Radiology, University Hospital Heidelberg, Heidelberg, Germany; 5 Department of Medicine V, Multiple Myeloma Section, University Hospital Heidelberg, Heidelberg, Germany; 6 National Center for Tumor Diseases (NCT), University Hospital Heidelberg, Heidelberg, Germany

## Abstract

**Objective:**

To investigate the reproducibility of size measurements of focal bone marrow lesions (FL) in MRI in patients with monoclonal plasma cell disorders under variation of patient positioning and observer.

**Methods:**

A data set from a prospective test–retest study was used, in which 37 patients with a total of 140 FL had undergone 2 MRI scans with identical parameters after patient repositioning. Two readers measured long and short axis diameter on the initial scan in *T*_1_ weighted, *T*_2_ weighted short tau inversion recovery and diffusion-weighted imaging sequences. The first reader additionally measured FL on the retest-scan. The Bland–Altman method was used to assess limits of agreement (LoA), and the frequencies of absolute size changes were calculated.

**Results:**

In the simple test–retest experiment with one identical reader, a deviation of ≥1 mm / ≥2 mm / ≥3 mm for the long axis diameter in *T*_1_ weighted images was observed in 66% / 25% / 8% of cases. When comparing measurements of one reader on the first scan to the measurement of the other reader on the retest scan, a change of ≥1 mm / ≥3 mm / ≥5 mm for the long axis diameter in *T*_1_ weighted images was observed in 78% / 21% / 5% of cases.

**Conclusion:**

Small deviations in FL size are common and probably due to variation in patient positioning or inter-rater variability alone, without any actual biological change of the FL. Knowledge of the uncertainty associated with size measurements of FLs is critical for radiologists and oncologists when interpreting changes in FL size in clinical practice and in clinical trials.

**Advances in knowledge:**

According to the MY-RADs criteria, size measurements of focal lesions in MRI are now of relevance for response assessment in patients with monoclonal plasma cell disorders.

Size changes of 1 or 2 mm are frequently observed due to uncertainty of the measurement only, while the actual focal lesion has not undergone any biological change.

Size changes of at least 6 mm or more in *T*_1_ weighted or *T*_2_ weighted short tau inversion recovery sequences occur in only 5% or less of cases when the focal lesion has not undergone any biological change.

## Introduction

Under current guidelines, whole-body MRI (wb-MRI) plays a major role for risk stratification, staging, and response assessment in monoclonal plasma cell disorders.^1–7^ In addition to the number of focal bone marrow lesions in MRI, the size of focal bone marrow lesions and their development has recently been shown to correlate with risk of progression in smoldering multiple myeloma (SMM),^8^ with progression-free survival and overall survival in multiple myeloma (MM),^9,10^ with advance of local tumour biology,^11^ and with development of osteolytic lesions corresponding to the respective focal lesion.^12^ According to MY-RADS,^13^ the evolution of the size of focal lesions is one criterion for the response assessment categories. Additionally, according to the updated IMWG guideline,^3^ a growing focal lesion in a patient with SMM now leads to upstaging to MM with indication of systemic treatment before end-organ damage occurs.^14^ Beyond MM, size measurements of bone (marrow) metastasis in wb-MRI also gain importance. It has been reported that bone metastasis are measurable in MRI and that RECIST guidelines might need to be adapted,^15^ and EORTC^16^ and MET-RADS-P^17^ recommendations recommend the size measurements of bone lesions for follow-up assessment in metastatic bone disease.

In summary, changes in focal bone marrow lesion size are now a decisive criterion, both in monoclonal plasma cell disorders and metastatic bone disease. However, no cut-off values have yet been defined to decide whether a measured change in lesion size is within the margin of measurement error and the lesion should be considered stable, or whether the measured change reflects an actual change of the biology of the disease and the lesion should be considered increasing/decreasing in size. The absence of knowledge regarding the reproducibility of size measurements of focal bone marrow lesions in wb-MRI, caused by inter-rater variability and patient positioning, leaves the radiologist with a crucial problem when making clinical decisions, as it is unclear whether a measured change in size actually reflects a biological change or is within the margin of measurement error.

Therefore, the purpose of this study was to investigate size measurement reproducibility of focal bone marrow lesions in wb-MRI regarding: (I) variability in patient positioning in test–retest, (II) inter- and (III) intra-observer variability, and (IV) a combined scenario in which both rater and patient positioning are varied.

## Methods and patients

### Patients

129 consecutive patients who underwent wb-MRI for proven or suspected monoclonal plasma cell disorder (MPCD) at Centre 1 (*n* = 48) or at Centre 2 (*n* = 81) between 30 November 2020, and 16 February 2021, were offered to participate in a prospective, institutional review board-approved (S-796/2020) imaging study, which had the primary goal to investigate radiomic feature repeatability and reproducibility in multicentric settings.^17^ Inclusion criteria were proven or suspected MPCD, written informed consent and age of at least 18 years. Patients were excluded in case of implants without dedicated MRI safety information from the respective manufacturer, claustrophobia, or poor clinical condition. Detailed information on inclusion and exclusion process including the consort flow diagram for the overall prospective study have been published elsewhere,^18^ and an abbreviated flowchart from the overall cohort to the examinations included in this analysis is provided in Supplementary Figure 1Supplementary Material 1. Patient characteristics of the included patients in this analysis are reported in Table 1. This study was conducted in line with the Declaration of Helsinki and all patients provided written informed consent before inclusion.

Supplementary Material 1.

**Table 1.** Patient characteristics for the study cohort

Characteristic	Number (proportion)
Female sex	11 (30%)
Age (median, range)	59 years (40–70 years)
**Ig-subtype**	
IgG κ	12 (32%)
IgG λ	10 (27%)
IgA κ	4 (11%)
IgA λ	4 (11%)
Light-chain κ	5 (14%)
Asecretory	1 (3%)
n.a.	1 (3%)
**Stage**	
Suspected MPCD	1 (3%)
MGUS	3 (8%)
SMM	4 (11%)
NDMM	3 (8%)
MM under / after systemic therapy	24 (70%)
**Tumor load**	
Patients with FLs in field of view	19
Mean number (±SD) of FLs in field of view per patient	6.7 (±10.1)
FLs in right pelvis	58
FLs in left pelvis	42
FLs in sacral bone	25
FLs in lumbar spine or femora (as included in field of view)	15
Mean long axis diameter (±SD) of FLs (*T*_1_W, Rater 1)	12.9 mm (±8.0 mm)
Mean short axis diameter (±SD) of FLs (*T*_1_W, Rater1)	8.6 mm (±4.1 mm)

FLs, focal bone marrow lesions; Ig, immunoglobulin; MGUS, monoclonal gammopathy of unknown significance; MM, multiple myeloma; MPCD, monoclonal plasma cell disorder; NDMM, newly diagnosed multiple myeloma; SD, standard deviation; SMM, smoldering multiple myeloma; *T*_1_W, *T*_1_ weighted; n.a, not available.

### Study design

**Figure 1.**
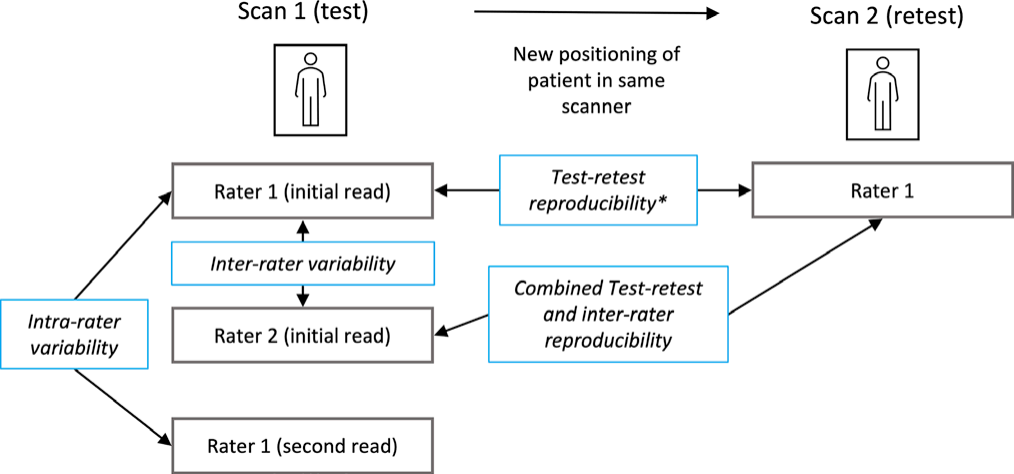
Study design regarding acquired MRI scans, study reads, and comparisons of measurements assessed in this study. In addition to the first scan (Scan 1), a second scan after new positioning of the patient (Scan 2) was performed. Study reads are given in grey boxes, comparisons are shown in blue boxes. *The test–retest reproducibility is also affected by the intrarater variability.

Details on the workflow regarding patient recruitment and measurements of this prospective study have been published before.^18^ Within this secondary analysis of a prospectively acquired data set, paired test–retest MRIs (‘scan 1’, ‘scan 2’) of the pelvic bone marrow before and after patient repositioning were acquired, using the same MRI scanner and same sequence parameters for both measurements (Figure 1). Details on patient repositioning are reported in Supplementary Material 1. A schematic overview over the different comparisons for which measurement variability was assessed is shown in Figure 1.

### Imaging

Imaging was performed with a 1.5 Tesla MRI Scanner (Siemens Magnetom Aera, Siemens Healthineers, Erlangen, Germany) with software version syngo MR E11. For whole-body scans, one ‘Head/Neck 20’ coil, two ‘Body 18’ coils and one ‘Peripheral Angio Feet 36’ coil were used. For the scans of the pelvic bone marrow, one ‘Body 18 coil’ was used. Imaging sequences comprised coronal *T*_1_ weighted (*T*_1_W) turbo spin echo, coronal *T*_2_ weighted (*T*_2_W) short-tau inversion recovery, and axial diffusion-weighted imaging (DWI). Further details are reported in Supplementary Material 1.

### Image assessment

Focal bone marrow lesions were defined as focal *T*_1_W-hypointensity with focal *T*_2_W-hyperintensity and focal hyperintensity in the b800 DWI, with at least 5 mm size. The longest diameter (referred to as ‘long axis diameter’) and the diameter perpendicular to the long axis diameter (referred to as ‘short axis diameter’) were measured in integer mm in *T*_1_W, *T*_2_W and b800 DWI images on a dedicated Picture Archiving and Communication System workstation (Centricity 4.2, GE Healthcare, Chicago, IL). Reader 1 (6 years of experience in reading scans with MPCDs) performed an initial read of the first scan and the retest scan side by side, in which he defined all FL to be included in this study and performed his initial measurements for both scans. Further information is reported in Supplementary Material 1. To assess the intra-rater variability, after a wash-out period of 5 months Reader 1 performed a second read of Scan 1, blinded to his earlier results. Reader 2 (fourth year of training) performed measurements of all FL on Scan 1, blinded to results obtained by Reader 1. When measuring a FL in all three sequences, readers were not blinded to their measurements of this FL from the other sequences, but were instructed to measure the longest diameter of the FL in each sequence independent from their prior measurements of this FL from the other sequences.

### Statistical analysis

To assess agreement of size measurements of FLs by different readers, different MRI-sequences or different patient positioning, LoA^19^ were computed, where the narrower the limits the better the agreement between the measurements. Visual presentation of the results obtained using the above approach was done using Bland–Altman plots (B&A plots).^19^ Proportion of absolute differences within pre-specified limits were also calculated. Statistical analysis was performed using R (v. 4.0.3, package nlme, R Foundation for Statistical Computing, Vienna, Austria). Further details are reported in Supplementary Material 1.

## Results

### Study cohort and acquired measurements

An abbreviated flowchart for the inclusion and exclusion process of patients and measurements used for this study is given in Supplementary Figure 1, and the full flowchart for patient inclusion and exclusion of the prospective study has been published before.^18^ 37 patients underwent MRI measurements before and after new positioning on the MRI couch, and 19 of these had FL in the field of view and thereby effectively contributed to this analysis. The overall data set comprised 140 FL, with the number of FL per patient ranging from 0 to 32. Patient characteristics of the study cohort are reported in Table 1. The study cohort included patients with an asymptomatic precursor stage of myeloma, patients with newly diagnosed MM, and patients which were receiving or had received systemic therapy. All test–retest measurements were performed on the same day.

**Figure 2.**
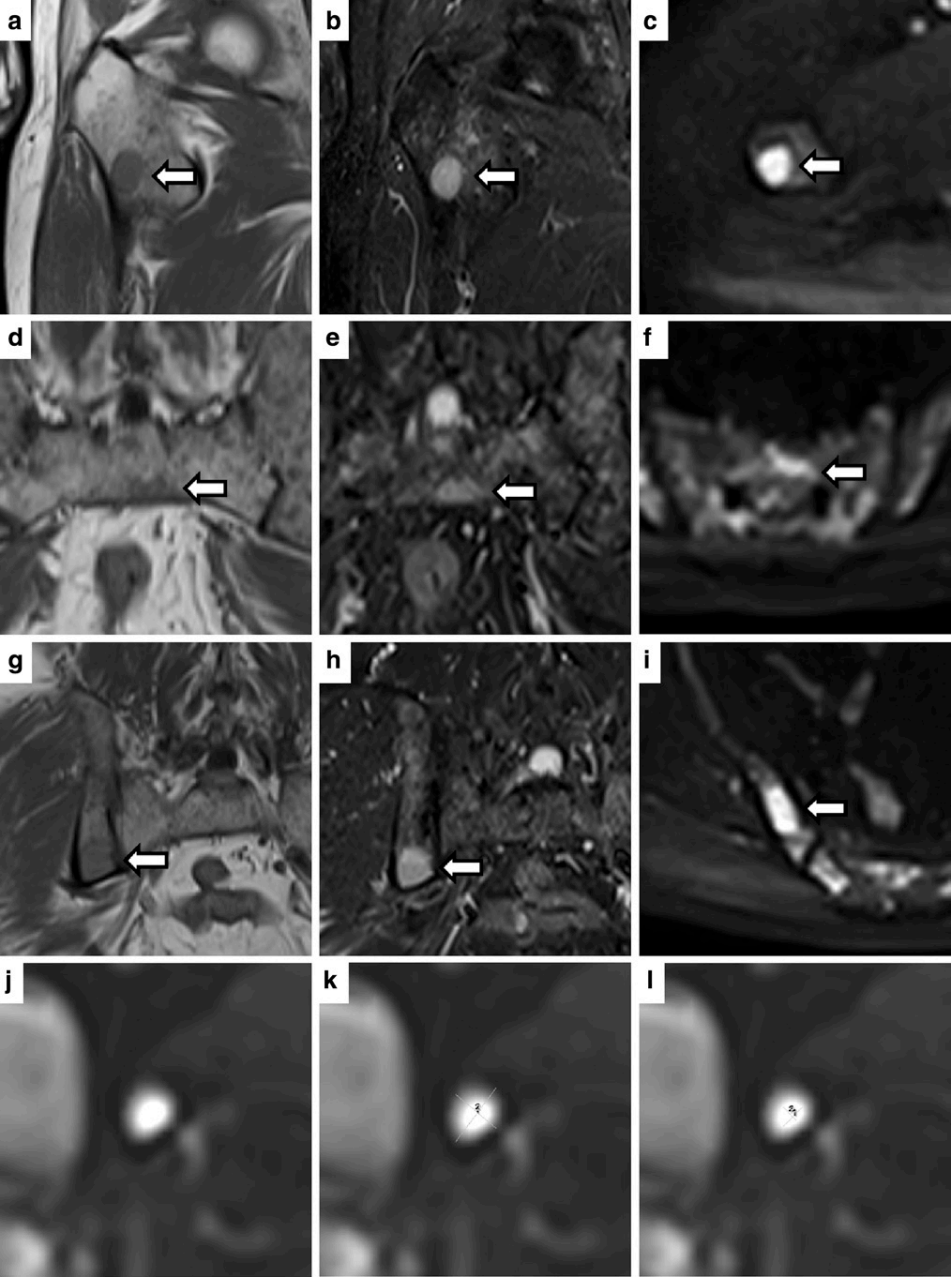
Exemplary cases from the data set displaying well-defined focal lesions *vs* cases causing differences in size measurements in the different scenarios. a, b and c show a well-circumscribed, clearly delimitable focal lesion in the right proximal femur in coronal *T*_1_W (**a**),coronal *T*_2_W (**b**) and axial b800 DWI images (**c**). d, e and f show a very irregularly shaped focal lesion in the sacral bone, which borders to the cortical bone on the ventral/caudal border and is irregularly defined on the other borders on all coronal *T*_1_W (**d**), coronal *T*_2_W (**e**) and axial b800 DWI image (**f**), which is also in part due to an adjoining micronodular/diffuse infiltration. g, h, and i show a focal lesion with a longitudinal oval shape in the right iliac bone. In the coronal *T*_1_W (**g**) and *T*_2_W (**h**) image, the lesion seems to have a circular shape, and was measured as 18 x 15 mm in *T*_1_W and 19 x 18 mm in *T*_2_W by Reader 1 on Scan 1. In the axial b800 DWI image (**i**), the focal lesion shows an oval shape, and was measured as 31 x 16 mm by Reader 1 on Scan 1. (**j**) shows a focal lesion in the left acetabulum in an axial high-b-value image, which had undergone cystic transformation after therapy. While the lesion has a high contrast to the surrounding tissue, the lesion shows a broad margin. In **(k**), a simulated measurement is shown which includes the complete margin into the measurement, giving diameters of 18 x 14 mm, while in **(l**) only the central core of the lesion without any of the margin is included, giving a measurement of 8 x 5 mm. Regarding the size measurement in the high-b-value image of the lesion depicted in **(j–l**), Rater 1 measured 15 x 12 mm in the initial read, 15 x 13 mm in his second read 5 months later, and 17 x 13 mm for this lesions on the second scan (retest), while Reader 2 measured this lesions as only 10 x 9 mm in his assessment of the initial scan. *T*_1_W, *T*_1_ weighted; T_2_W, *T*_2_ weighted; DWI, diffusion-weighted imaging.

Besides the quantitative results displayed below, a few general observations are to be reported from the study reads. The delimitability varied between different FL (Figure 2a–f). Oval configured FL lead to markedly different assessments between measurements from coronal and axial orientation in few cases (Figure 2g–i). Especially, cystically transformed focal lesions often showed a wide ‘brightness margin’, in which readers had relevant scope to define how much of the margin to include in the measurement (Figure 2j–l).

### Reproducibility in test–retest after patient repositioning

**Figure 3.**
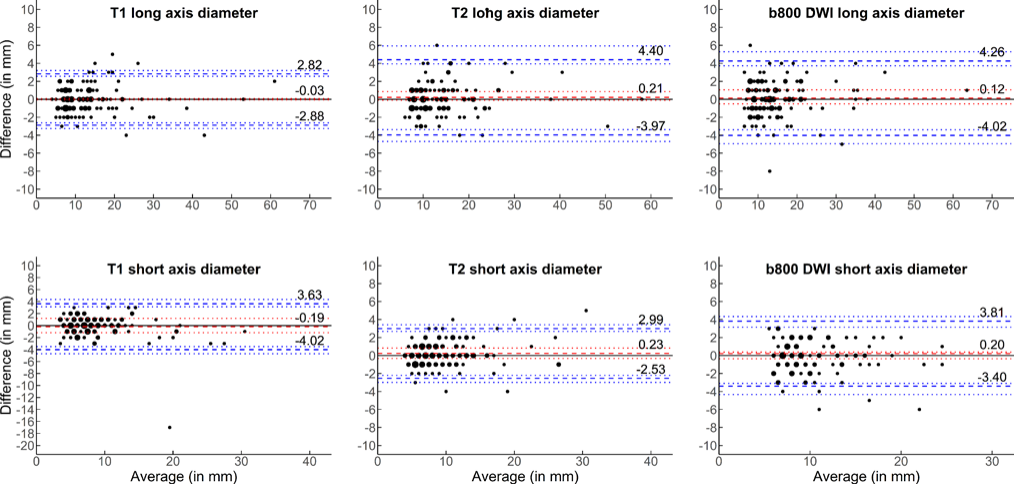
Reproducibility of size measurements of focal lesions in test–retest after patient repositioning. Bland–Altman plots are shown for the size measurements of the long and short axis of focal lesions in *T*_1_W, *T*_2_W and b800 DWI sequences. The average of both measurements in millimetre (mm) is given on the x-axis and the absolute difference between both measurements in mm is given on the y-axis. The dashed red line represents the bias, with the dotted red lines representing the 95% confidence intervals. The dashed blue lines represent the lower and upper limits of agreement, with corresponding 95% confidence intervals represented by the dotted blue lines. The size of points is proportional to the number of respective observations. *T*_1_W, *T*_1_ weighted; T_2_W, *T*_2_ weighted; DWI, diffusion-weighted imaging.

B&A plots for the measurements on the initial scan and the repeated scan after patient repositioning, both assessed by Reader 1, are shown in Figure 3, and metrics on bias and LoA are displayed in Table 2. There was no marked bias in any comparison. LoAs were up to 4.4 mm for *T*_1_W and *T*_2_W sequences and up to 4.3 mm for b800 DWI sequences.

**Table 2.** Overview over reproducibility of measurements in test–retest, inter-, intra-rater, and a combination of test–retest and inter-rater setting

Comparison scenario/variable	Bias (mm)	adjusted *p*-value	LoA (mm)
**Test–retest**			
*T*_1_W long axis	−0.03	<0.001	−2.88, 2.82
*T*_1_W short axis	−0.19	0.417	−4.02, 3.63
*T*_2_W long axis	0.21	0.618	−3.97, 4.40
*T*_2_W short axis	0.23	0.007	−2.53, 2.99
b800 DWI long axis	0.12	0.618	−4.02, 4.26
b800 DWI short axis	0.20	0.598	−3.40, 3.81
Inter-rater			
*T*_1_W long axis	0.06	0.800	−5.26, 5.37
*T*_1_W short axis	−0.49	0.256	−5.04, 4.05
*T*_2_W long axis	0.41	0.193	−5.07, 5.90
*T*_2_W short axis	0.37	0.417	−4.04, 4.78
b800 DWI long axis	2.63	<0.001	−3.75, 9.01
b800 DWI short axis	1.67	<0.001	−3.34, 6.67
Intra-rater			
*T*_1_W long axis	0.36	0.417	−4.13, 4.86
*T*_1_W short axis	0.26	0.632	−4.61, 5.12
*T*_2_W long axis	0.11	0.123	−4.38, 4.59
*T*_2_W short axis	−0.12	0.457	−3.29, 3.06
b800 DWI long axis	0.92	0.256	−5.83, 7.67
b800 DWI short axis	−0.21	0.598	−5.04, 4.62
**Combined variation of patient positioning and change of rater**			
*T*_1_W long axis	0.56	0.330	−5.17, 6.29
*T*_1_W short axis	−0.24	0.618	−5.52, 5.04
*T*_2_W long axis	0.25	0.256	−5.59, 6.09
*T*_2_W short axis	0.37	0.457	−3.94, 4.67
b800 DWI long axis	2.12	<0.001	−4.24, 8.49
b800 DWI short axis	1.57	<0.001	−3.73, 6.87

DWI, diffusion-weighted imaging; LoA, limits of agreement; *T*_1_W, *T*_1_ weighted; *T*_2_W, *T*_2_ weighted.

Table 3 reports the frequency with which magnitudes of size changes were observed in the test–retest scenario when both measurements were performed by the same reader (Reader 1).

**Table 3.** Frequency of absolute changes of size measurements of the long axis diameter and the short axis diameter under variation of patient positioning

Magnitude of change of long axis diameter (positive or negative, in mm)	Frequency (in %) in		
	*T*_1_W	*T*_2_W	b800 DWI
≥1 mm	66.2	71.9	71.0
≥2 mm	25.2	27.3	38.4
≥3 mm	7.9	15.1	16.7
≥4 mm	3.6	7.2	8.0
≥5 mm	0.7	1.4	2.9
**Magnitude of change of short axis diameter (positive or negative, in mm)**	**Frequency (in %) in**		
	***T*_1_W**	***T*_2_W**	**b800 DWI**
≥1 mm	65.5	66.2	71.7
≥2 mm	25.2	22.3	41.3
≥3 mm	7.9	7.2	12.3
≥4 mm	0.7	3.6	3.6
≥ 5 mm	0.7	0.7	2.1

DWI, diffusion-weighted imaging ; *T*_1_W, *T*_1_ weighted; *T*_2_W, *T*_2_ weighted.

### Inter- and intra-rater variability

**Figure 4.**
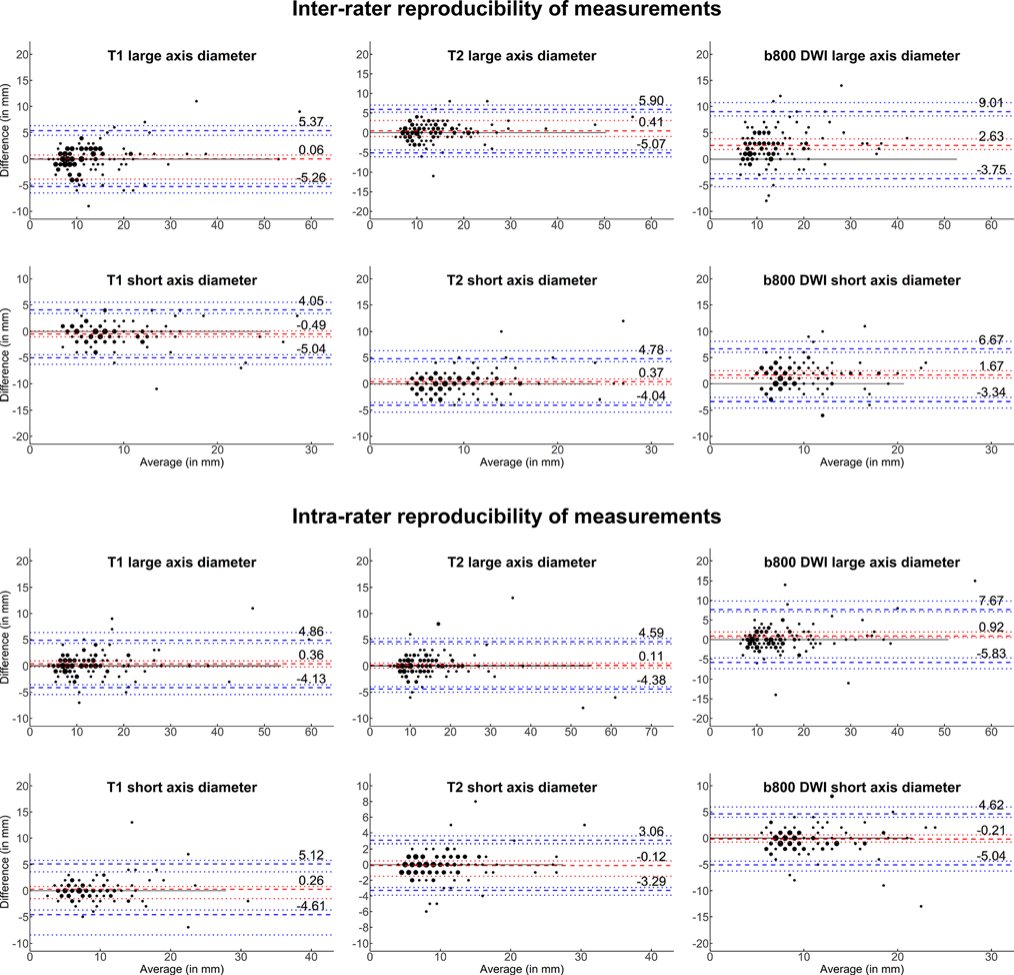
Reproducibility of size measurements of focal lesions regarding inter- and intra-rater variability. Bland–Altman plots are shown for the size measurements of the long and short axis of focal lesions in *T*_1_W, *T*_2_W and b800 DWI sequences. The average of both measurements in millimetre (mm) is given on the x-axis and the absolute difference between both measurements in mm is given on the y-axis. The dashed red line represents the bias, with the dotted red lines representing the 95% confidence intervals. The dashed blue lines represent the lower and upper limits of agreement, with corresponding 95% confidence intervals represented by the dotted blue lines. The size of points is proportional to the number of respective observations. *T*_1_W, *T*_1_ weighted; T_2_W, *T*_2_ weighted; DWI, diffusion-weighted imaging.

Figure 4 displays the B&A plots for the inter- and intra-rater comparisons. Metrics on bias and LoAs are displayed in Table 2. There was no marked bias in the intra-rater comparisons, and LoAs up to 5.1 mm for *T*_1_W/*T*_2_W and up to 7.7 mm for the b800 DWI sequences. In the inter-rater comparison, there was also no bias for *T*_1_W and *T*_2_W sequences, however in b800 DWI images there was a small, statistically significant bias of 2.6 mm/1.6 mm for the long axis/short axis measurement (both *p* < 0.001). The LoA were up to 5.9 mm for *T*_1_W/*T*_2_W images and up to 9.0 mm for the b800 DWI sequences.

### Measurement reproducibility under both variation of patient positioning and change of rater

**Figure 5.**
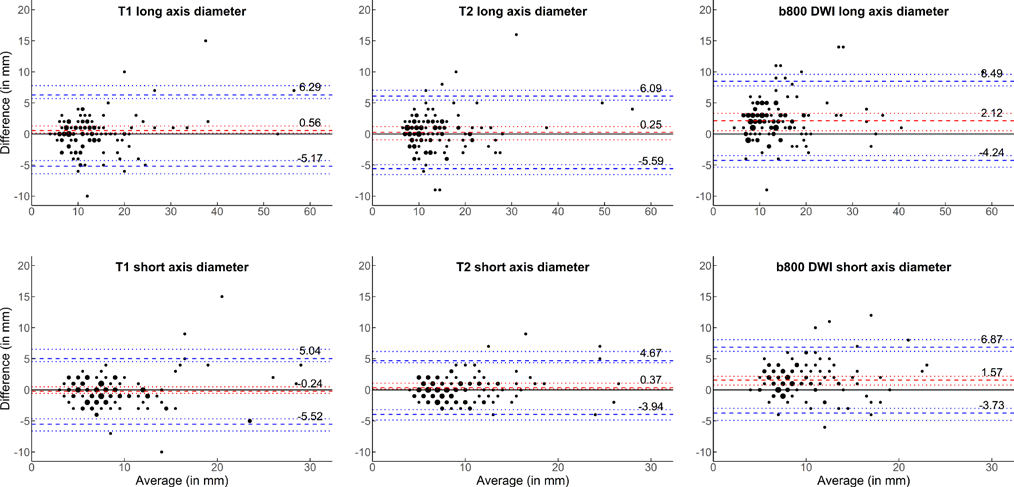
Reproducibility of size measurements of focal lesions under variation of both patient positioning and reader. Bland–Altman plots are shown for the size measurements of the long and short axis of focal lesions in *T*_1_W, *T*_2_W and b800 DWI sequences. The average of both measurements in millimetre (mm) is given on the x-axis and the absolute difference between both measurements in mm is given on the y-axis. The dashed red line represents the bias, with the dotted red lines representing the 95% confidence intervals. The dashed blue lines represent the lower and upper limits of agreement, with corresponding 95% confidence intervals represented by the dotted blue lines. The size of points is proportional to the number of respective observations. *T*_1_W, *T*_1_ weighted; T_2_W, *T*_2_ weighted; DWI, diffusion-weighted imaging.

After investigating the influence of each factor separately in the previous analyses, Figure 5 displays the B&A plots for the comparison of the size measurements of Reader 2 on the initial scan to the measurements of Reader 1 on the retest scan, representing a concomitant variation of both patient positioning and reader. While for *T*_1_W and *T*_2_W measurements no bias was observed, there was a statistically significant bias of 2.1 mm/1.6 mm for b800 DWI (both *p* < 0.001). *T*_1_W and *T*_2_W measurements had LoAs of up to 6.3 mm, while b800 DWI measurements had LoA of up to 8.5 mm.

Table 4 depicts the frequency of different size changes found in the scenario of combined change of rater and change of patient positioning.

**Table 4.** Frequency of absolute changes of size measurements of the long axis diameter and the short axis diameter under combined variation of both patient positioning and rater

Magnitude of change of long axis diameter (positive or negative, in mm)	Frequency (in %) in		
	*T*_1_W	*T*_2_W	b800 DWI
≥1 mm	71.2	80.6	91.4
≥2 mm	40.3	45.3	66.9
≥3 mm	25.2	27.3	46.8
≥4 mm	14.4	15.1	24.5
≥5 mm	9.4	8.6	19.4
≥6 mm	5.0	5.0	10.8
≥7 mm	3.6	4.3	7.9
≥8 mm	2.2	3.6	7.2
**Magnitude of change of short axis diameter (positive or negative, in mm)**	**Frequency (in %) in**		
	***T*_1_W**	***T*_2_W**	**b800 DWI**
≥1 mm	77.7	73.4	82.7
≥2 mm	44.6	38.8	56.1
≥3 mm	20.9	13.7	35.3
≥4 mm	9.4	6.5	20.1
≥5 mm	5.0	2.9	10.1
≥6 mm	2.9	2.2	7.2
≥7 mm	2.9	2.2	3.6

DWI, diffusion-weighted imaging; *T*_1_W, *T*_1_ weighted; *T*_2_W, *T*_2_ weighted.

## Discussion

In most oncologic entities, follow-up tumour size measurements are central to evaluate the course of disease and steer treatment accordingly.^20^ In monoclonal plasma cell disorders, over recent years, the value of size measurements of FL in MRI has also been demonstrated in different contexts,^8,9,11,12,21^ and also beyond MPCD, size measurements of bone (marrow) metastasis are increasingly recommended.^15,16,22^ The MY-RADS criteria^13^ now incorporate the changes of size of FL to define response assessment categories (RAC) and the IMWG recommendations now incorporate growth of a focal lesion as a criterion for upstaging to MM and start of systemic treatment.^3^ However, no distinct cut-off values have yet been defined to determine whether a FL should be considered stable or decreasing/increasing in size. Both in clinical practice and in research assessments, it is critical for radiologists and oncologists to decide whether a measured change in size reflects an actual biological change of the FL, or whether the change in size is just caused by differences in patient positioning or measurement by the reader. In the current study, we performed different experiments on a prospectively acquired test–retest data set to determine the reproducibility of size measurements of FLs in whole-body MRI.

In a clinical setup, a radiologist will perform lesion measurements on both the current scan and previous ones to detect either tumour shrinkage or growth, but even minor variance in patient positioning may influence the results. In our experiment with the patient being repositioned, we found that with a single reader the measured long axis diameter would deviate by 1 mm/2 mm/3 mm, depending on the sequence, in approximately 70, 30 and 10%, even though the lesion itself was exactly the same and did not show any biological change.

In most study assessments, a radiologist will measure the size of lesions and these measurements will be recorded in a database at the respective time point. When the study results are analysed, measurements from different readers between different time points will be compared. Again, the position of the patient has changed randomly between the time points. This scenario is best captured by our experiment with a combined variation of patient positioning and rater. In this scenario, we found that changes of the size of the long axis diameter of 1 mm/3 mm/5 mm occurred in approximately 80, 33 and 12%, while the FL was exactly identical.

In summary, changes of 1–2 mm are frequently observed even though there is no change in the actual lesion, while changes of ≥6 mm in *T*_1_W and *T*_2_W sequences are observed only rarely—in 5% of cases or less—when the lesion is identical. Awareness regarding these levels of uncertainty is crucial when interpreting follow-up wb-MRI examinations in MPCD, both in clinical practice and when interpreting imaging data in trials.

From the other experiments, we further conclude that size measurements should primarily be performed on *T*_1_W and *T*_2_W sequences instead of high-b-value DWI sequences, as these measurements show better reproducibility, with generally narrower LoA and no relevant bias between different scenarios or readers. Our results thereby support a former expert recommendation that size measurements should be performed on *T*_1_W sequences,^13,22^ however size measurements on *T*_2_W sequences would be equally appropriate. B800 DWI images are very valuable for lesion detection and detection of diffuse infiltration^23–25^ and can provide measurements on tissue characteristics as ADC, which have been shown to be of value in MM.^26–30^ However, spatial resolution of DWI at least in our current protocol is lower compared to morphologic sequences, and borders of lesions are relatively ill-defined due to partial volume effects. Based on our observations from individual cases, we further recommend using the same orientation of images for all follow-up measurements, as some FLs are configurated in a way that might lead to markedly different assessments when measuring in different orientations. Automatic FL segmentations would allow for objective size assessment without the subjective influence of a human reader. While recently automatic algorithms for bone marrow segmentation in whole-body MRIs have been developed,^31,32^ to the best of our knowledge, an algorithm for automatic focal lesion segmentation is not yet available.

Several prior studies have assessed the reproducibility of different imaging characteristics in MPCDs. Lecouvet and colleagues reported that the intra- and inter-reader agreement for detecting bone marrow involvement in MRI was very good.^33^ Messiou and colleagues investigated the interobserver agreement regarding a myeloma disease burden score and found excellent interobserver agreement for wb-MRI.^34^ This group also reported that interobserver agreement for the disease burden score in wb-MRI was superior to the interobserver agreement for wb-CT^35^ and investigated the inter- and intraobserver agreement of ADC and fat fraction measurements from FLs.^36^ However, these studies investigated variation caused by observers only, and no test–retest measurements were performed, and the studies cannot inform about the uncertainty which is caused by patient positioning and scan acquisition. A recent study investigated the repeatability and reproducibility of bone marrow ADC measurements in patients with MPCDs, regarding readers, patient positioning, MRI sequences, different MRI scanners with 1.5 and 3T.^18^ An additional study by Elgendy and colleagues has investigated the repeatability of ADC measurements using 3T scanners, measuring ADC both in focal lesions and diffuse infiltration.^37^ The current study complements the prior literature on reproducibility of imaging characteristics in MPCDs regarding the very central aspect of size measurements of FL.

Due to high cost of scan time for MRI and due to additional radiation exposure for patients in CT, prospective test–retest studies are difficult to perform and generally relatively sparse throughout oncologic entities. Oxnard and colleagues investigated test–retest reproducibility of size measurements in CT in patients with lung tumours and reported the LoA to be −4.8 mm to +4.8 mm, with a median tumour size of 3.7 cm.^38^ Zhao and colleagues also performed a prospective test–retest study and reported relative LoAs of agreement around ± 20 percent for all three readers, with a mean tumour diameter of approx. 3.5 cm.^39^ In comparison to these two studies, the lesions in the present study were markedly smaller, and the resolution and slice thickness differs between wb-MRI and chest CT. Furthermore, we assume that the delimitability of lung tumours is better than the delimitability of focal lesions in the bone marrow. Therefore, it is plausible that the reproducibility of size measurements of FL in MRI is different than that of lung tumours in chest CT.

A limitation of the current study is the limited number of patients. However, while in prior studies there was only one tumour per patient,^38,39^ in the current study a total of 140 FLs were assessed. A second limitation is that our assessment relies on test–retest scans of pelvic bone marrow only, however, we assume that the results generalise well to the rest of the skeleton. A third limitation is that we did not include contrast enhanced sequences, despite the fact that a recent publication reported that post-contrast dixon sequences are superior for lesion identification.^40^

## Conclusion

This study provides insights on the reproducibility of size measurements of focal bone marrow lesions in MRI caused by both variations in patient positioning and variations of radiological assessment by different readers. Awareness of the measurement uncertainty is crucial for radiologists and oncologists when deciding whether the measured change in size during follow-up reflects an actual biological change of the malignancy, pointing to disease progression or therapy response, or whether a change in size only lies within the domain of uncertainty associated with the measurement. These results about size measurement uncertainty should be taken into account when expert committees define cut-off-values for therapy response assessments based on FL size. We further conclude that preferably *T*_1_W or *T*_2_W sequences instead of DWI sequences should be used for size measurements of focal lesions in MRI due to their superior reproducibility.
