# Supplementary material for: Test–retest, inter- and intra-rater reproducibility of size measurements of focal bone marrow lesions in MRI in patients with multiple myeloma
Source: Br J Radiol. 2023 Apr 12;96(1145):20220745. doi: 10.1259/bjr.20220745 (PMC10161907; doi:10.1259/bjr.20220745)
Supplement: Supplementary Material 1. [file bjr.20220745.suppl-01.docx]

# Test-retest, Inter- and Intra-rater reproducibility of size measurements of focal bone marrow lesions in MRI in patients with multiple myeloma

**- Supplements -**

**Details on patient repositioning**

Patients left the MRI couch between the scans and were instructed to walk a couple of steps. They were additionally offered to stretch, use the toilet, drink some water, or sit down for a couple of minutes before undergoing the second scan. Then, patients were newly positioned on the MRI couch and the coil for imaging the pelvic bone marrow was newly positioned, as it would be positioned when performing a whole-body scan.

**Details on imaging sequences**

Imaging sequences comprised coronal T1-weighted turbo spin echo (T1w; repetition time 528ms, echo time 8.4ms, in plane resolution 1.3mm x 1.3mm, slice thickness 5.0mm, 10% distance factor), coronal T2-weighted short-tau inversion recovery (T2w; repetition time 3650, echo time 56ms, in plane resolution 0.7mm x 0.7mm (interpolated), slice thickness 5.0mm, 10% distance factor, fat suppression: slice-selective inversion recovery (inversion time=160ms)), and axial diffusion weighted imaging (DWI; Diffusion-EPI iShim, repetition time 5130, echo time 64ms, in plane resolution 1.8mm x 1.8mm (interpolated), slice thickness 6.0 mm, 0% distance factor, b values: 50s mm^-2^ and 800s mm^-2^, fat suppression: slice-selective inversion recovery (inversion time=180ms)). Full details on the MRI scanners, software version and MRI sequence parameters have been reported before ^1^ and can be found there as scanner 1, protocol 1a.

**Further details on image assessment**

The field of view when performing the scans for this study was primarily set to the pelvis, but focal lesions in the lower lumber spine or proximal femora were also measured if fully covered in the field of view in all three sequences. Lesions which were very ill-defined, for example due to very low contrast to surrounding diffuse infiltration or hypercellular bone marrow due to therapy effects, were excluded in consensus with an experienced senior reader (20 years of experience in reading scans with MPCDs).

**Further details on statistical analysis**

Due to the multiple nature of the measurements of a patient with each rater/MRI-sequence, the LoA as well as the mean bias were computed using a mixed effects model to take into account all the sources of variability. We employed the approach detailed in Parker et al. ^2^ where the paired differences were computed and modelled directly, with the patient as a random effect. For the estimation of 95% confidence intervals for the LoA and mean bias, we employed the parametric bootstrap-t approach as in Parker et al. ^2^. Both absolute differences and relative differences after log-transformation were computed. As relative differences showed funnel-shaped configuration for long and short axis diameters, absolute scale was more appropriate for our data, and consequently absolute differences are reported in the manuscript. Test on whether the bias was significantly different from zero was performed using a linear mixed model. P-values are reported, adjusted for multiple testing with the Bonferroni-Holm method.

**Supplementary figure 1.** Abbreviated flow chart for patient inclusion and exclusion process, as well as measurements included in the current analysis. The full inclusion / exclusion process from the prospective Multiscanner, Multiprotocol test-retest-study has been published elsewhere (compare main text reference 27).

**References for Supplement:**

1. Wennmann M, Thierjung H, Bauer F, Weru V, Hielscher T, Grözinger M, et al. Repeatability and Reproducibility of ADC Measurements and MRI Signal Intensity Measurements of Bone Marrow in Monoclonal Plasma Cell Disorders: A Prospective Bi-institutional Multiscanner, Multiprotocol Study. Invest Radiol [Internet]. 2022;57(4). Available from: https://journals.lww.com/investigativeradiology/Fulltext/2022/04000/Repeatability_and_Reproducibility_of_ADC.8.aspx

2. Parker RA, Weir CJ, Rubio N, Rabinovich R, Pinnock H, Hanley J, et al. Application of Mixed Effects Limits of Agreement in the Presence of Multiple Sources of Variability: Exemplar from the Comparison of Several Devices to Measure Respiratory Rate in COPD Patients. PLoS One. 2016;11(12):e0168321.
